# Annual cycle variations in the gut microbiota of migratory black-necked cranes

**DOI:** 10.3389/fmicb.2025.1533282

**Published:** 2025-02-07

**Authors:** Yujia Zhang, Ruifeng Ma, Suolangduoerji, Shujuan Ma, Akebota Nuertai, Ke He, Hongyi Liu, Ying Zhu

**Affiliations:** ^1^College of Animal Science and Veterinary Medicine, Southwest Minzu University, Chengdu, Sichuan, China; ^2^College of Grassland Resources, Institute of Qinghai-Tibetan Plateau, Sichuan Provincial Forest and Grassland Key Laboratory of Alpine Grassland Conservation and Utilization of Qinghai-Tibetan Plateau, Southwest Minzu University, Chengdu, Sichuan, China; ^3^Sichuan Ruoergai Wetland National Nature Reserve Administration, Ruoergai, Ruoergai, Aba Tibetan and Qiang Autonomous Prefecture, China; ^4^Luxian NO.1 High School, Luzhou, Luzhou, Sichuan, China; ^5^College of Animal Science and Technology, College of Veterinary Medicine, Zhejiang A & F University, Hangzhou, China; ^6^The Co-Innovation Center for Sustainable Forestry in Southern China, College of Life Sciences, Nanjing Forestry University, Nanjing, China

**Keywords:** black-necked crane, annual cycle, gut microbiota, migratory birds, high-altitude

## Abstract

**Introduction:**

Migratory birds exhibit unique annual cycles that complicate their gut microbiota. However, the annual dynamics of gut microbiota in migratory birds remain unclear, hindering our understanding of their environmental adaptation.

**Methods:**

Here, we collected fecal samples from black-necked cranes (*Grus nigricollis*) across four seasons at their breeding grounds and used wintering ground data from databases to characterize their gut microbial compositions throughout the year.

**Results and discussion:**

The results showed that the gut microbiota was clustered by season (Bray-Curtis: *R*^2^ = 0.348, *p* < 0.001; UniFrac: *R*^2^ = 0.352, *p* < 0.001). And the summer samples exhibited higher alpha (Simpson and Shannon), beta diversity (Bray-Curtis and UniFrac) and more diverse functions in gut microbiota compared to other seasons. Furthermore, in summer, the gut microbiota exhibited several balanced relative abundances at the family level, whereas *Lactobacillaceae* family dominated during the other seasons. Thirty-six ASVs were identified by random forest analysis to distinguish samples from distinct seasons. Despite having greater diversity, the summer gut microbiota had a simpler network structure than the other seasons (fewer edges and nodes). The dispersal limitation during random processes also significantly influenced gut microbial community assembly. Overall, the gut microbiota of the black-necked crane undergoes dynamic adjustments to adapt to seasonal environmental changes, which may be associated with the variations in diet across seasons. These results enhance our understanding of the gut microbiota of wild migratory birds and support further research on black-necked cranes.

## 1 Introduction

Gut microbes form complex symbiotic relationships with their hosts (Nichols and Davenport, 2021). The gut microbiota is influenced by host conditions (Bajinka et al., 2020) and plays an important role in maintaining gut health and host metabolic pathways (Claus et al., 2011; Lee et al., 2022). Wild animals face more complex environmental changes (e.g., seasonal diet and elevation) than animals in captivity, and they undergo physiological and behavioral adjustments to adapt (Dallas and Warne, 2023). Research on wild animals has revealed that environmental changes influence the gut microbiota. For example, alpha and beta gut microbiota diversity increases with habitat elevation in pikas (*Ochotona curzoniae*) (Li H. et al., 2019). Furthermore, studies on Tibetan macaques (*Macaca thibetana*) (Xia et al., 2021) and ground squirrels (*Spermophilus dauricus*) (Yang et al., 2021) have revealed that their gut microbiota clusters by season. Additionally, during seasons of food abundance, the gut microbiota of animals exhibits higher diversity to meet the demands of digesting a diverse range of foods (Sun et al., 2016).

Migratory birds have a unique annual cycle (Schmiedová et al., 2023), and their periodic migrations between breeding and wintering grounds expose them to diverse challenges (Lu et al., 2022). Because of their exposure to complex and variable environments, wild migratory birds have emerged as pivotal models for investigating microbial-host interactions (Elzinga et al., 2019). During migration, birds must adapt to their different habitats and local food resources (Grond et al., 2018). The gut microbiota plays a crucial role in bird migration and habitat changes (Zhang F. et al., 2020). The gut microbiota assists birds in breaking down plant fibers and detoxifying harmful substances in their diet (Drovetski et al., 2019; Waite and Taylor, 2015; Zhang et al., 2021). During the cold season, bacteria such as Firmicutes become more abundant, facilitating energy intake (Liukkonen et al., 2024; Yao et al., 2023). Similarly, during migration, microbes associated with fat deposition, such as *Corynebacterium*, increase in abundance (Skeen et al., 2023; Thie et al., 2022; Zhang et al., 2021). However, the unique annual cycle of birds makes their gut microbiota complex and difficult to study (Wu et al., 2018).

Previous studies have identified seasonal variations in the diversity and functional composition of gut microbes in migratory birds. These include white-headed cranes (*Antigone vipio*) (Dong et al., 2021), black-winged stilts (*Himantopus himantopus*), black-tailed godwits (*Limosa limosa*), and redshanks (*Tringa totanus*) (Zhang et al., 2021). Some migratory birds have extended breeding or wintering periods, and a prolonged stay in one location can lead to changes in the gut microbiota. This has been observed in studies of wild relict gulls (*Larus relictus*) (Yao et al., 2023) and the great bustard (*Otis tarda*) (Lu et al., 2024).

Most studies on the microbiota of migratory birds have focused primarily on a single period of the annual cycle. However, understanding gut microbial changes throughout the annual cycle can provide valuable insights into the relationship between gut microbes and their hosts under varying environmental conditions, thereby aiding in the conservation of wild and rare avian species (Song et al., 2014).

The black-necked crane (*Grus nigricollis*) is a lifelong highland bird that is currently listed as threatened by the IUCN. High-altitude environments impose environmental pressures (e.g., hypoxia, low temperature, and high ultraviolet light) on animals (Liu et al., 2022), and consequently, birds inhabiting these environments adapt their physiological state and gut microbiota accordingly (Wang et al., 2020). For example, the Eurasian tree sparrow (*Passer montanus*) enlarges its digestive organs (Sun et al., 2023), whereas the Himalayan bluetail (*Tarsiger rufilatus*) enriches its gut with *Lactobacillus* and *Pseudomonas* to aid food metabolism (Zhang et al., 2024). Each year, black-necked cranes migrate from their wintering grounds (e.g., Yunnan-Kweichow Plateau, the southern slopes of the Himalayas) to their breeding grounds (Qinghai–Tibet Plateau, Xinjiang) in March and return in November (Gao et al., 2007; Wang et al., 2013). They have a relatively long breeding period (8 months) and a shorter wintering period (4 months) (Pu and Guo, 2023). Black-necked cranes primarily inhabit farmland areas during the winter and feed predominantly on wetlands during the breeding season in Zoige, China (Dong et al., 2016). As black-necked cranes transition from the growing season to the non-growing season in Zoige, they encounter different food resources. These seasonal differences, along with the differences between breeding and wintering periods, provide an excellent opportunity to study host-gut microbial interactions throughout the annual cycle of a migratory bird.

In this study, black-necked crane wintering data from previous studies were used in conjunction with fecal samples collected during the breeding period across the four seasons in Zoige. The resulting data were analyzed using 16S rRNA gene sequencing to determine the gut microbiota community structures of black-necked cranes throughout their annual cycle. We hypothesized that different seasons have different gut microbial community structures. Black-necked cranes’ gut microbiota would respond to the seasonal variation in food resources, showing higher diversity during seasons of food abundance.

## 2 Materials and methods

### 2.1 Sample collection

**Figure 1:**
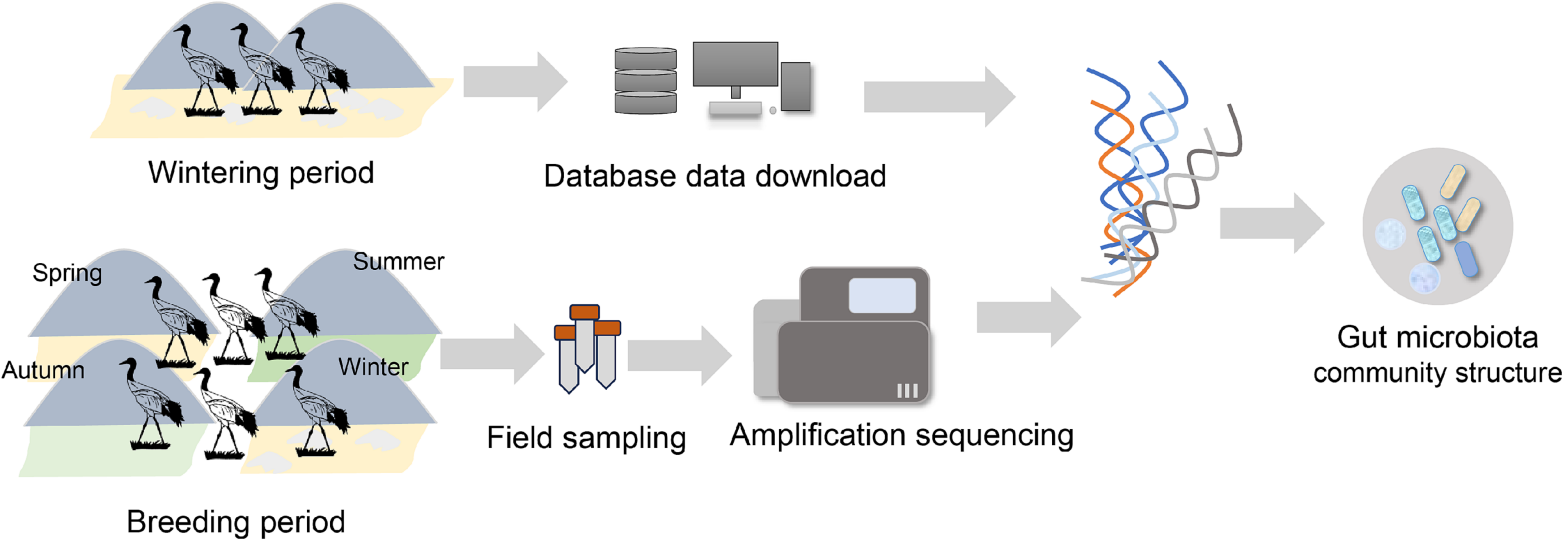
Overview of our study design.

Black-necked cranes are the only species known to inhabit and reproduce on high plateaus throughout their life. The Zoige Wetland National Nature Reserve in China is an important breeding site for black-necked cranes, whereas Caohai and Dashanbao in China are important wintering sites. Black-necked cranes primarily inhabit meadows or marsh meadows in Zoige (Bai et al., 2022), which is one of the hotspots for biodiversity, with plants from the Cyperaceae, Ranunculaceae, and Asteraceae families having the largest number of species. The Zoige area also supports a rich diversity of animal species, including amphibians, fish, and various arthropods such as Diptera and Coleoptera (Xiang et al., 2009). Furthermore, we found arthropods are main animal-deprived food of the black-necked crane’s diet in our previous study (Ma et al., 2024). However, in Caohai and Dashanbao during the wintering period, black-necked cranes primarily inhabit farmland areas (Wu et al., 2013), where they feed on grains, potatoes, and some invertebrates (Dong et al., 2016). We collected black-necked crane feces from 19 locations in Zoige in April and September 2022 as well as in July and November 2023 (spring: *N* = 30, summer: *N* = 30, autumn: *N* = 30, winter: *N* = 30). The spring and autumn samples are part of a dataset associated with a recently published paper (Ma et al., 2025). During sample collection, we observed black-necked cranes feeding for approximately 2–3 h and collected feces after the birds had left. Using sterile toothpicks, we extracted the internal portion of each fecal sample and placed it into a 15 mL centrifuge tube. The samples were stored in liquid nitrogen and sent to a laboratory in Chengdu, China. We also downloaded the gut microbiome data of black-necked cranes for the wintering period from the National Center of Biotechnology Information (NCBI; project numbers PRJNA681985) (Dashanbao; Zhao et al., 2021), PRJNA992803, and PRJNA995432 (Caohai; Wang et al., 2024). In total, 41 winter samples were obtained from the database (Supplementary Table S1; Figure 1).

### 2.2 Gut microbiota detection

Fecal DNA was extracted using an OMEGA Soil DNA Kit (M5635-02; Omega Bio-Tek, Norcross, GA, United States). Negative controls were used for extraction and amplification, and no detectable products were observed. The 16S rRNA region (V3–V4) of the gut microbiota was detected using the primers 338F/806R (Lee et al., 2012). The polymerase chain reaction (PCR; 25 μL) contained: 5 × reaction buffer 5 μL, 5 × GC buffer 5 μL, dNTP (2.5 mM) 2 μL, forward primer (10 uM) 1 μL, reverse primer (10 uM) 1 μL, DNA template 2 μL (20 ng/μL), ddH_2_O 8.75 μL, and Q5 DNA polymerase 0.25 μL. The amplification program was as follows: initial denaturation at 98°C for 2 min, denaturation at 98°C for 15 s, annealing at 55°C for 30 s, extension at 72°C for 30 s for 30 cycles, and final extension at 72 for °C 5 min. A DNA library was constructed using a TruSeq Nano DNA LT Library Prep Kit (Illumina). Paired-end sequencing of the 16S rRNA gene was conducted using an Illumina NovaSeq 6,000 platform at Personal Bio (Shanghai Personal Biotechnology Co., Ltd., Nanjing, China). The easyAmplicon pipeline^1^ was used to process the sequencing data. We used the “fastx_filter” function of VSEARCH (v2.14.1) to trim primers and perform quality filtering. The “derep_fulllength” function of VSEARCH was employed for the dereplication task, with a minimum unique size of 135. To denoise, we used the unoise3 function of USEARCH (v10.0.240), and the “usearch_global” function of VSEARCH was used to generate an amplicon sequence variant table. Rarefaction analysis was conducted using “alpha_div_rare” in USEARCH, and we did not observe obvious batch effects based on cluster dendrogram and PCA analysis (Supplementary Figure S1).

### 2.3 Statistical analysis

#### 2.3.1 Alpha and beta diversity

All statistical tests were conducted using R software (version 4.2.1, 2022). USEARCH was employed to calculate the alpha diversity measures, including the Shannon and Simpson indices, as well as the beta diversity metrics, which comprised the Bray-Curtis distance and the weighted UniFrac distance, for the microbiota analysis. Constrained ordination (partial canonical analysis of principal coordinates, CAP) and unconstrained ordination (nonmetric multidimensional scaling, NMDS) were performed to evaluate seasonal effects based on the Bray–Curtis distance and weighted UniFrac distance. For the CAP, we conducted permutational multivariate analysis of variance (PERMANOVA) and analysis of variance (ANOVA) to validate its significance using 999 permutations in the “vegan” v2.6.4 (Oksanen et al., 2007) package. CAP was performed using the “ordinate” function in the “phyloseq” v1.42.0 package (Hu L. et al., 2018). For NMDS, seasonal effects were detected using the analysis of similarities (ANOSIM) function in the “vegan” package with 999 permutations. NMDS was performed using the “metaMDS” function in the “vegan” package. To investigate the effect of season on the Shannon index, Simpson index, Bray–Curtis distance, and weighted UniFrac distance, we modeled season as a fixed factor, location and sample collection year as a random factor using “lme4” v 1.1.33 (Bates et al., 2014). We applied transformations using the “powerTransform” function from the “car” package (v3.1.2) (Fox et al., 2007) when the normality or constant variance of model residuals was not met. The indices that required transformation included the Simpson index, Bray–Curtis distance, and weighted UniFrac distance.

#### 2.3.2 Differential analysis of gut microbiota between seasons

We used a random forest model to distinguish bacterial taxa between seasons, employing the machine learning algorithm in the “randomForest” v4.7.1.1 package (Breiman, 2001). The seasonal classification model was trained on 70% of the dataset. Error rates were estimated at the phylum, class, order, family, and genus levels, and the taxon level was selected to obtain the cross-validation error curve, as described in our previous study (Zhu et al., 2024).

#### 2.3.3 Co-occurrence network of gut microbiota

A co-occurrence network was used to illustrate gut microbiota interactions at the family level. Spearman correlations among all samples were calculated and corrected for compositionality effects using 1,000 bootstrap iterations and permutations with the “ccrepe” package (v 1.38.1). *p*-values were adjusted for multiple testing using the default Benjamini–Hochberg–Yekutieli method, retaining values with an adjusted *p* < 0.05. To investigate the seasonal effects on topological properties, we extracted sub-networks of individual samples using the “subgraph” function in the “igraph” package by specifying individual vertices (Csardi and Nepusz, 2006). The number of edges, nodes, average degrees, and modularity were used to evaluate the complexity of the black-necked crane gut microbiota network. We used a generalized linear mixed model with a Poisson distribution for the number of edges and nodes, which are count data. For average and modularity, we employed a generalized linear mixed model with binomial error in the “lmer4” package. The sampling season was considered a fixed factor, and the sampling location, sample collection year was considered a random factor.

#### 2.3.4 Community assembly of gut microbiota

The Nearest Taxon Index (βNTI) was used to qualitatively evaluate the deterministic or stochastic processes of community assembly, using the “picante” package (v 1.8.2). If the βNTI is >2 or < −2 this indicates that the microbiota community is affected by the deterministic assembly process. However, if the βNTI is > −2 and < 2, this indicates that the gut microbiota community is impacted by a stochastic process. Phylogenetic-bin-based null model analysis (iCAMP) was also conducted using the “iCAMP” package (v 1.5.12) to examine the assembly mechanisms of different gut microbiota groups in black-necked cranes. The iCAMP results identified five assembly mechanisms: dispersal limitation, drift and others, heterogeneous selection, homogeneous selection, and homogenizing dispersal.

#### 2.3.5 Prediction of gut microbiota function

We used PICRUSt2 (Douglas et al., 2020) to predict the functional profiles of microbial communities across all samples on the basis of the 16S rRNA gene. The Shannon index, Simpson index of function were calculated using “vegan” package and we modeled season as a fixed factor, location and sample collection year as a random factor using “lme4” v 1.1.33 (Bates et al., 2014).

## 3 Results

We obtained 10,949,408 high-quality reads from 161 samples (breeding: 8775868, wintering: 2173540), with an average of 68008.75 reads per sample. Rarefaction analysis revealed that the sequencing data captured most of the gut microbiota from each black-necked crane fecal sample (Supplementary Figure S2). In total, 18 phyla, 36 classes, 65 orders, 132 families, and 233 genera were identified.

### 3.1 Gut microbiota diversity of the black-necked crane between seasons

**Figure 2:**
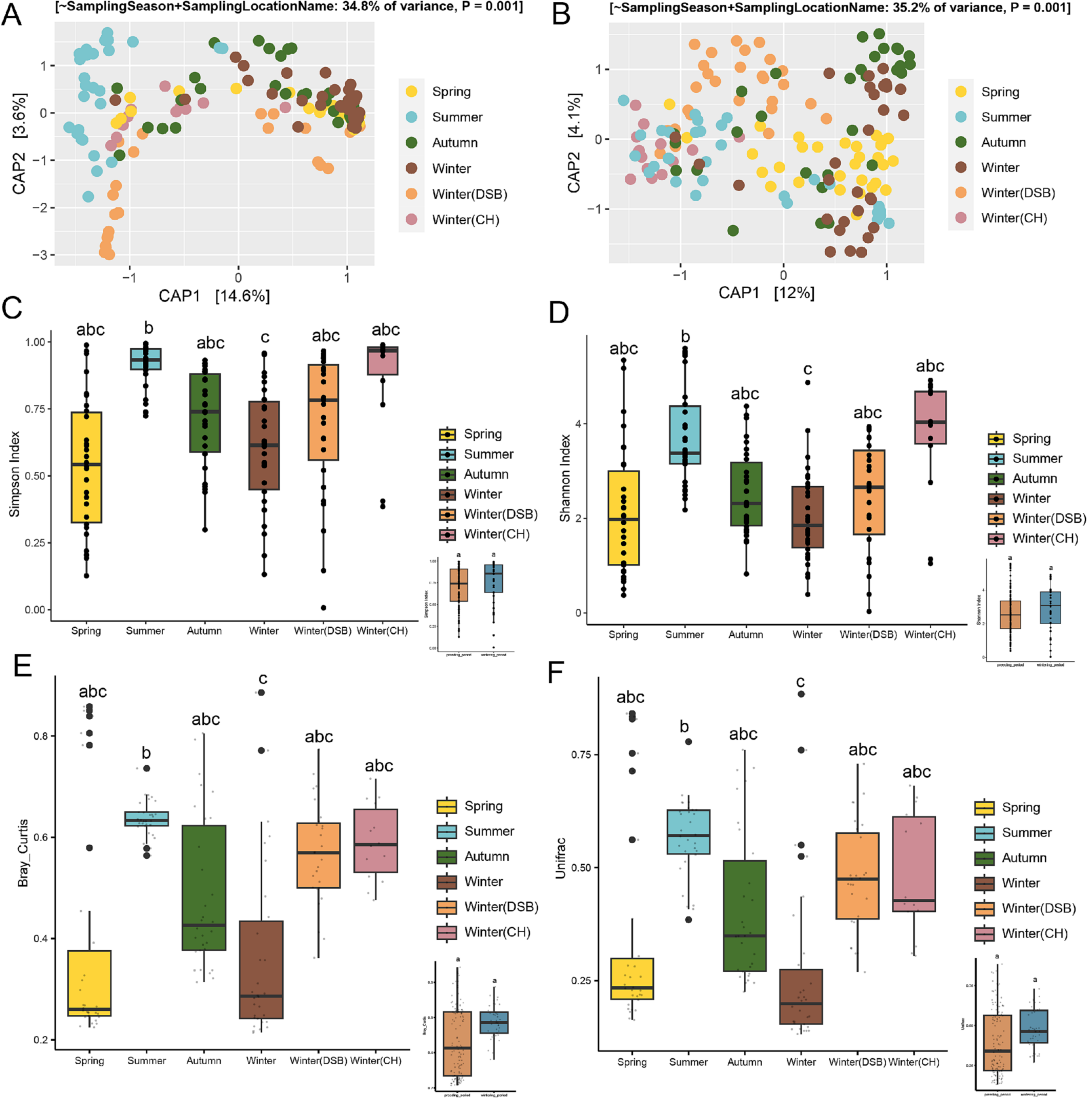
Seasonal variability in the gut microbiota of the black-necked crane. The constrained ordination (CAP) was based on **(A)** Bray-Curtis and **(B)** UniFrac distances. Alpha diversity of the gut microbiota was measured using **(C)** Simpson and **(D)** Shannon indices, with the inset plots showing alpha diversity between the breeding and wintering periods. Beta diversity of the gut microbiota was based on **(E)** Bray-Curtis and **(F)** UniFrac distances, with the inset plots illustrating beta diversity between the breeding and wintering periods. Different colors represent different seasons.

The constrained ordination analysis (CAP) showed that gut microbiota exhibited a seasonal pattern (ANOVA and PERMANOVA, Bray-Curtis: *R*^2^ = 0.348, *p* < 0.001, UniFrac: *R*^2^ = 0.352, *p* < 0.001; Figures 2A,B), and the unconstrained ordination (NMDS) analysis revealed the same seasonal pattern (ANOVA, Bray-Curtis: *R* = 0.295 *p* < 0.001, UniFrac: *R* = 0.274, *p* < 0.001) based on Bray-Curtis and weighted UniFrac distances (Supplementary Table S2). They were relatively dispersed across different seasons (Figures 2A,B).

The alpha-diversity during the wintering period was higher than that during the breeding period. There were significant differences among the seasons (Simpson: χ^2^ = 57.719, *p* < 0.001; Shannon: χ^2^ = 24.494, *p* < 0.001; Figures 2C,D; Supplementary Table S3). The Simpson indices for the summer and winter Caohai samples were higher than those for the other seasons (Figure 2C; Supplementary Table S4). The Shannon index also indicated that the overwintering Caohai samples had higher diversity than the spring samples (Figure 2D; Supplementary Table S4).

Beta-diversity analysis showed that the wintering period had higher diversity than the breeding period, but the difference was not significant (Figures 2E,F). There were significant differences among the seasons (Bray-Curtis: χ^2^ = 30.907, *p* < 0.001; UniFrac: χ^2^ = 41.594, *p* < 0.001).

Summer, CH, and DSB had the highest beta diversity, whereas winter had the lowest diversity based on both Bray-Curtis and UniFrac distance (Figures 2E,F; Supplementary Table S4).

### 3.2 Gut microbiota abundances and biomarkers between seasons

We observed variations in the relative abundances of the gut microbiota. Firmicutes were the dominant phylum in all groups, except for the winter (CH) group in which Proteobacteria were dominant (Supplementary Table S5).

**Figure 3:**
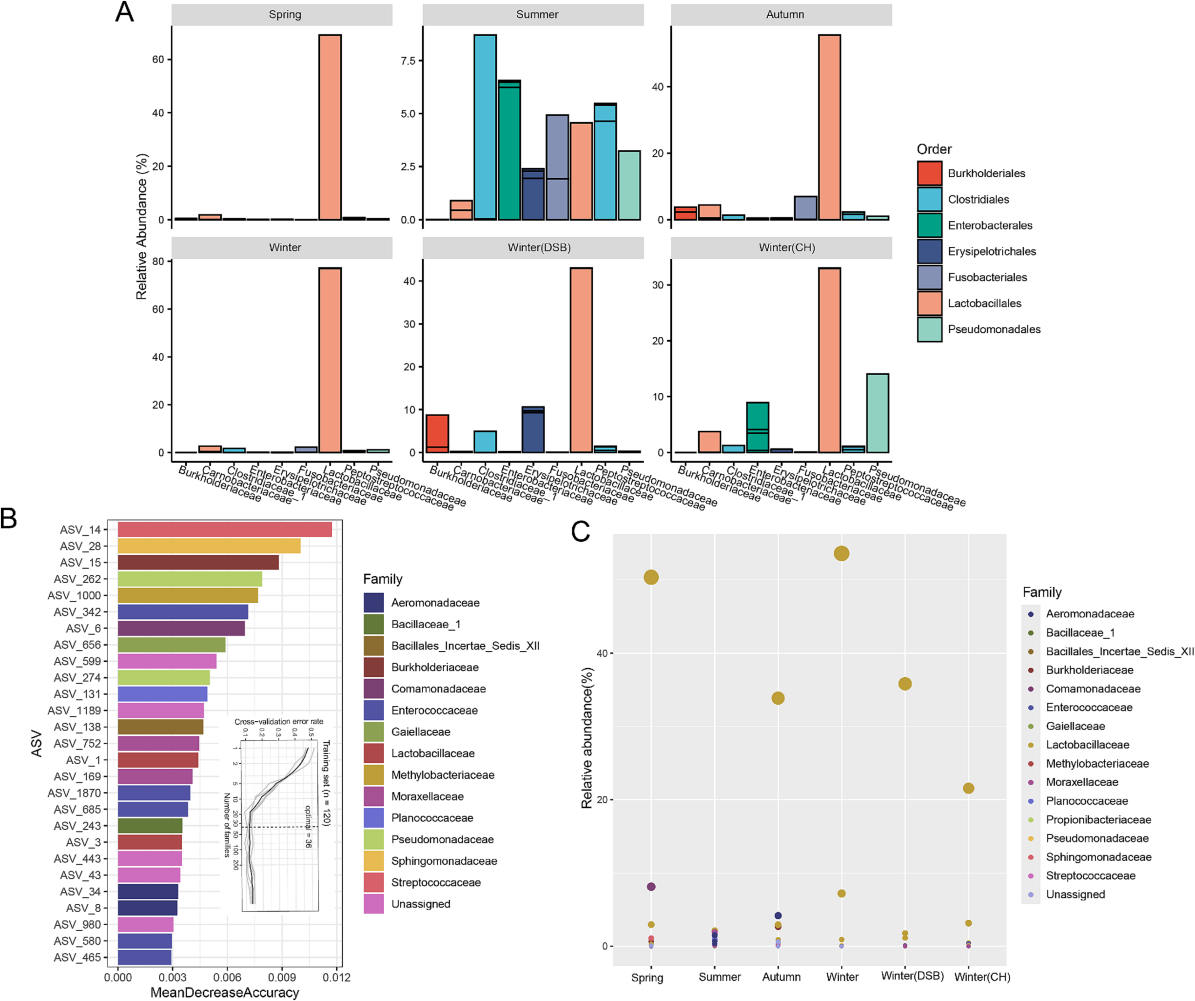
The dominant and distinct gut microbiota across seasons. **(A)** Normalized relative abundance of the most common genera in different seasons colored by order and separated at the top 10 family levels. Microbiota taxa were predicted using a random forest model for different seasons. **(B)** The top 36 microbiota ASVs were identified in the training set based on their relative abundance. **(C)** The abundance of microbiota across different seasons is shown, with bubble size representing abundance and color indicating family.

At the family level, *Lactobacillaceae* were the dominant microbiota (spring: 68.2%; summer: 4.8%; autumn: 54.9%; winter: 76.4%; DSB: 42.2%; CH: 27.3%; Figure 3A; Supplementary Table S6), except for during the summer when *Clostridiaceae_1* were dominant (8.7%; Supplementary Table S6). During the summer, certain other microorganisms exhibited relatively high abundances, such as *Pseudomonadaceae* (summer: 3.0%) and *Enterobacteriaceae* (summer: 7.2%; Figure 3A; Supplementary Table S6).

We further analyzed seasonal variations in gut microbiota biomarkers. The random forest-based model revealed that the ASV level provided the highest accuracy for classifying gut microbiota across different levels. The cross-validation error rate was 0.13 when using the 36 ASVs identified as having distinct microbiota (Figure 3B). The ASV of *Lactobacillaceae* was lower in abundance in the summer, whereas the ASVs of *Moraxellaceae* and *Planococcaceae* were higher (Figure 3C; Supplementary Figure S3).

### 3.3 Co-occurrence network of gut microbiota between seasons

**Figure 4:**
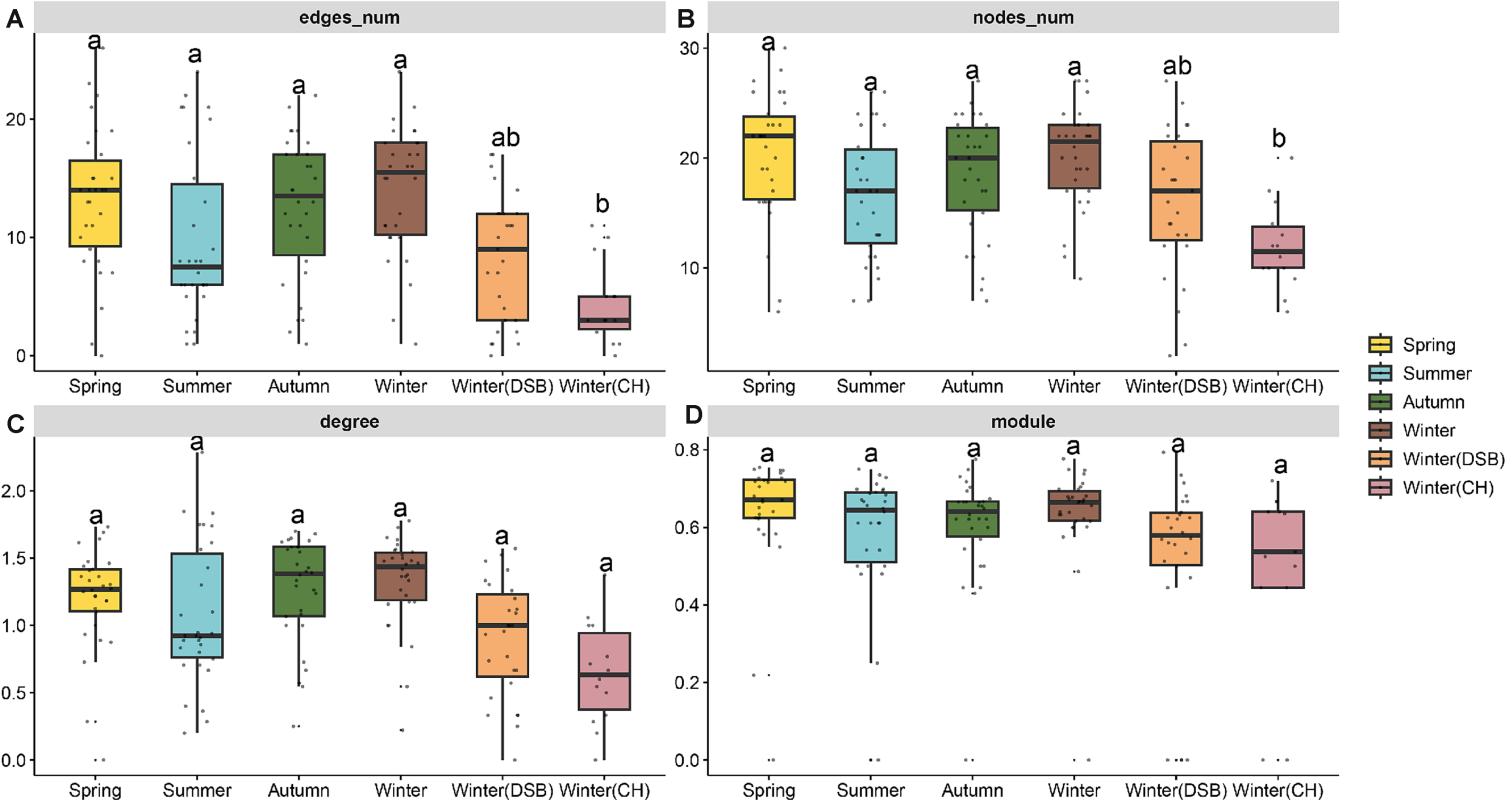
Topological properties of the co-occurrence network of black-necked crane gut microbiota in different seasons, **(A)** edges number, **(B)** nodes number, **(C)** average degree, and **(D)** modularity.

In total, 40 nodes (families) and 42 connections (edges) were retained in the black-necked crane co-occurrence network. Only the module did not significantly differ between seasons (Supplementary Table S7). The summer network topology was simpler, whereas the spring and winter exhibited more nodes, edges, and degrees. The nodes, edges, and degrees in winter (CH) and summer were lower than those in other seasons (nodes: χ^2^ = 33.144, *p* < 0.001, edges: χ^2^ = 36.183, *p* < 0.001, Figure 4; Supplementary Tables S7, S8).

### 3.4 Community assembly of black-necked crane gut microbiota

**Figure 5:**
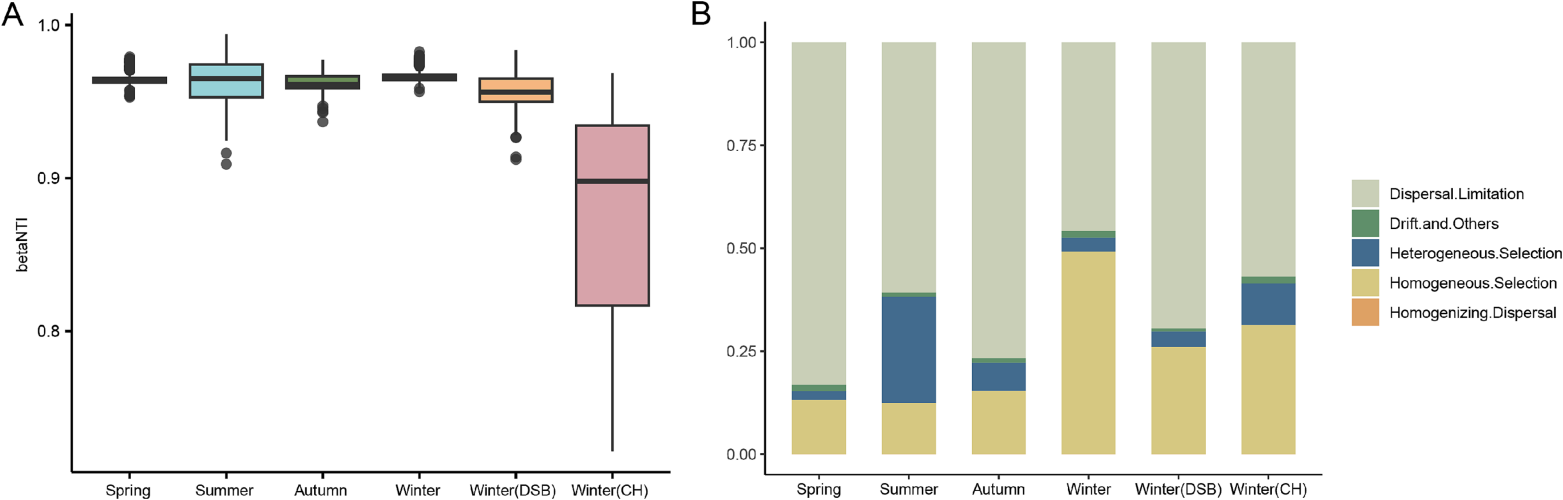
Importance of bacterial communities from different seasons in the gut microbiota of the black-necked crane. **(A)** The Nearest Taxon Index (βNTI) of the gut microbiota, −2 < βNTI <2 indicates that stochastic processes play a significant role in shaping the gut microbiota. **(B)** Relative importance of ecological processes for microbiota across different seasons.

The βNTI results showed that −2 < βNTI > 2, indicating that the stochastic process is an important factor influencing gut microbiota assembly across all seasons (Figure 5A). ICAMP analysis was performed to evaluate the gut microbiota assembly processes in the different groups. The dispersal limitation (spring: 0.792; summer: 0.612; autumn: 0.728; winter: 0.445; DSB: 0.548; CH: 0.704) was the major driver of gut microbiota assembly in all seasons except winter (Figure 5B; Supplementary Table S9). However, the results for winter contradict the βNTI findings, as homogeneous selection (winter: 0.503), a deterministic process, was the major driver of gut microbiota assembly (Figures 5A,B).

### 3.5 Functional prediction of black-necked crane gut microbiota

The functional profiles inferred by PICRUSt2 indicated significant variations in the Simpson index (χ^2^ = 13.368, *p* < 0.05), while the Shannon index did not differ among seasons (χ^2^ = 7.296, *p* > 0.05). Additionally, both the Simpson and Shannon indices were higher in the summer samples relative to other seasons (Supplementary Figure S4).

## 4 Discussion

Elucidating the changes in the gut microbiota of the migratory black-necked crane throughout its annual cycle is crucial for understanding its environmental adaptations. In this study, we examined the gut microbiota of the black-necked crane throughout its annual cycle and found differences in its composition, diversity, function, and co-occurrence networks. In most groups, stochastic processes were more important than deterministic processes in gut microbiota assembly.

### 4.1 Diversity and composition of gut microbiota in response to seasonal dietary changes

Food resources in the environment are crucial factors that influence gut microbiota (diversity and composition) (Scott et al., 2013). In our previous study on the dietary of black-necked cranes in Zoige, we found that a greater diversity of arthropods dietary in black-necked cranes in autumn than in spring (Ma et al., 2024). The current study revealed that a similar trend in the gut microbiota diversity, with the black-necked crane exhibiting lower alpha diversity in spring than in autumn. This suggests that there may be an association between the richness of diet and diversity of gut microbiota. Our findings imply that as dietary diversity increases, so does the diversity of the gut microbiota. Additionally, the current study revealed highest alpha and beta diversity of gut microbiota in summer (Figure 2), which leads us to speculate that the diet of black-necked cranes is most diverse during this period. The abundant water and heat resources in Zoige during summer, which contribute to rich food availability (Zhang Z. et al., 2020), likely support the hypothesis by providing a more varied array of food resources for the cranes. Our findings suggest a link between diet and microbiota diversity, prompting the need for future research on the relationship between dietary intake and gut microbiota composition.

Significant differences in gut microbiota composition have also been reported between seasons of food abundance and scarcity (Orkin et al., 2019). Studies on Tibetan macaques (*Macaca thibetana*) have shown that during seasons of food abundance, gut microbiota exhibit a higher level of diversity (Sun et al., 2016). To adapt to the abundance of food, the gut microbiota shifts and exhibits higher intra- and inter-species diversity (Zhao et al., 2023). The gut microbiota of the Greater Horseshoe Bats (*Rhinolophus ferrumequinum*) (Xiao et al., 2019) and Forest Musk Deer (*Moschus berezovskii*) (Hu X. et al., 2018) were also found to show higher diversity in summer, which has an abundance of food compared with other seasons. Our findings are consistent with those of previous studies. We also observed that the gut microbiota exhibited higher alpha diversity in Caohai and its wintering grounds than at the other locations tested. This could be because human-maintained fields provide ample food, similar to the abundance observed under natural summer conditions (Bergmann et al., 2015).

We found that some gut microbiota families were enriched only in summer (e.g., *Moraxellaceae*, *Planococcaceae*, *Bacillaceae_1*). *Moraxellaceae* is associated with the benzoate degradation pathway (Torrecillas et al., 2023), *Planococcaceae* can modulate valine production (Li, 2018; Wu et al., 2024), and *Bacillaceae_1* is linked to insect lipids (Li et al., 2022; Weththasinghe et al., 2022). Gut microbiota can rapidly respond to novel food components (Leeming et al., 2019). Previous studies have found that short-term dietary changes alter the gut microbiota of animals; however, these changes are difficult to observe after the return to a normal diet (Leeming et al., 2019). This enrichment likely reflects the animals’ need for diverse materials during digestion.

In summer, black-necked cranes had a more diverse microbiota and a lower relative abundance of dominant bacteria. However, during other seasons, the *Lactobacillaceae* family was dominant. An increase in a stable gut microbiota may represent an adaptation to cope with harsh environments (Jing et al., 2022; Santos et al., 2024). The *Lactobacillaceae* family’s strong adaptability allows for long-term colonization, maintenance of the intestinal barrier, and resistance to harmful bacteria, and helps hosts adapt to environmental changes (Santos et al., 2024). Extensive colonization by microorganisms ensures adequate energy intake (Ducarmon et al., 2019). The persistence of colonizing species in animals is likely due to their role in degrading storage carbohydrates, such as starch and fiber (Lee et al., 2024). *Lactobacillaceae*, known for their involvement in carbohydrate digestion, may colonize the gut for extended periods.

### 4.2 The gut microbiota network responds to seasonal changes

During summer, gut microbial samples revealed high microbial diversity but fewer nodes, edges, and degrees in the co-occurrence network, indicating a simpler network structure. This is likely due to the abundance of available food sources, which enables opportunistic bacteria to thrive and temporarily dominate (Stein et al., 2013). However, transient gut microbiota often have lower competitive adaptability in the gut than long-term colonizing species, which is why they do not persist (Lee et al., 2024). Once the season of food abundance has passed, these transient microbial changes are unlikely to persist. However, this situation is transient, and the complexity of the microbial network is expected to evolve.

We observed more complex microbial networks in other seasons, indicating that the microbiota networks had more nodes, edges, than those in summer. These complexities arise because of the harsh survival challenges that occur outside the summer. High environmental stress may cause the microbiota to establish more positive interactions within communities (Li G. et al., 2019) and support the stress-gradient hypothesis (Bertness and Callaway, 1994; Maestre et al., 2009). To overcome these difficulties, animals adjust their microbial networks to enhance their adaptability by increasing the complexity of their gut microbiota, which can be considered a strategy for biological adaptation to diverse environments (Faust and Raes, 2012). Adaptation has been found in many species, including wild ass (*Equus kiang*) (Gao et al., 2020), great tit (*Parus major*) (Bodawatta et al., 2021), Plateau Zokor (*Eospalax baileyi*) (Liu et al., 2024), and bharal (*Pseudois nayaur*) (Gao et al., 2024).

### 4.3 Stochastic processes are important for microbiota community assembly

Dispersal limitation was the primary driver of the gut microbiota assembly in black-necked cranes during all seasons except winter. Dispersal limitations are important for microbiota assembly. This pattern has been observed in studies on honeybees (*Apis cerana* and *Apis mellifera*) (Ge et al., 2021) and birds such as the common nightingale (*Luscinia megarhynchos*) (Sottas et al., 2021), thrush nightingale (*Luscinia luscinia*) (Sottas et al., 2021), and green-winged teal (*Anas crecca*) (Wang et al., 2022). Dispersal limitations reduce the ease with which gut microbes spread between individuals. Previous studies on mammalian gut microbes have found that geographical proximity enhances microbial communication among animals, whereas increased physical distance is a key factor affecting the composition of gut microbes (Moeller et al., 2017). Birds, with higher mobility and broader activity ranges than other animals, experience reduced gut microbiota interactions among individuals (Weinhold, 2022). The reduced interaction of the gut microbiota could be a significant factor affecting the gut microbial composition of black-necked cranes.

## 5 Conclusion

This study investigated the annual cycle of gut microbiota in migratory black-necked cranes. We found that the diversity, composition, predicted dominant functions and co-occurrence networks of the gut microbiota varied across seasons. The summer samples exhibited greater alpha diversity and beta diversity, as well as more diverse functions compared to other seasons. In all seasons except summer, *Lactobacillaceae* dominated the gut microbiota. The network structure of the gut microbiota was simpler in summer than in other seasons. Dispersal limitations were identified as a key factor influencing the assembly of gut microbial communities. Overall, black-necked cranes exhibit dynamic adjustments in their gut microbiota to adapt to annual environmental changes, which might be related to the variation of their seasonal diet. Our research reports on the gut microbiota of black-necked cranes throughout their annual cycle, providing valuable insights for the study of migratory birds’ gut microbiota. Future research should focus on multi-year continuous sampling, particularly incorporating samples collected during migration and pay greater attention to the relationship between diet and the gut microbiota of animals.

## Data Availability

The original contributions presented in the study are publicly available. This data can be found here: https://ngdc.cncb.ac.cn/gsa/s/qPJgx6YF.

## Footnotes

^1^ https://github.com/YongxinLiu/EasyAmplicon/blob/master/pipeline.sh
