# Supplementary material for: Annual cycle variations in the gut microbiota of migratory black-necked cranes
Source: Front Microbiol. 2025 Feb 7;16:1533282. doi: 10.3389/fmicb.2025.1533282 (PMC11844351; doi:10.3389/fmicb.2025.1533282)
Supplement: Supplementary file 2 [file Data_Sheet_1.docx]

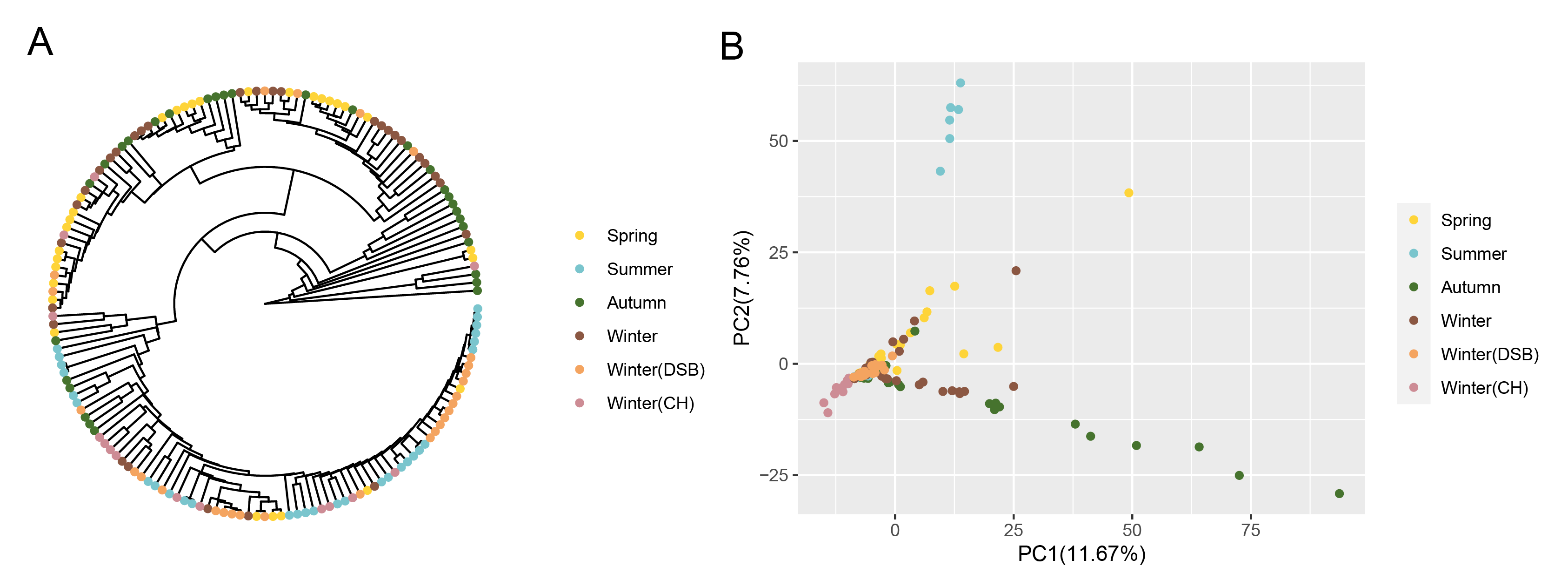


**Figure S1** The cluster dendrogram and the principal components analysis (PCA) demonstrated that the results were not influenced by the batch effect, with each batch corresponding to either different season.


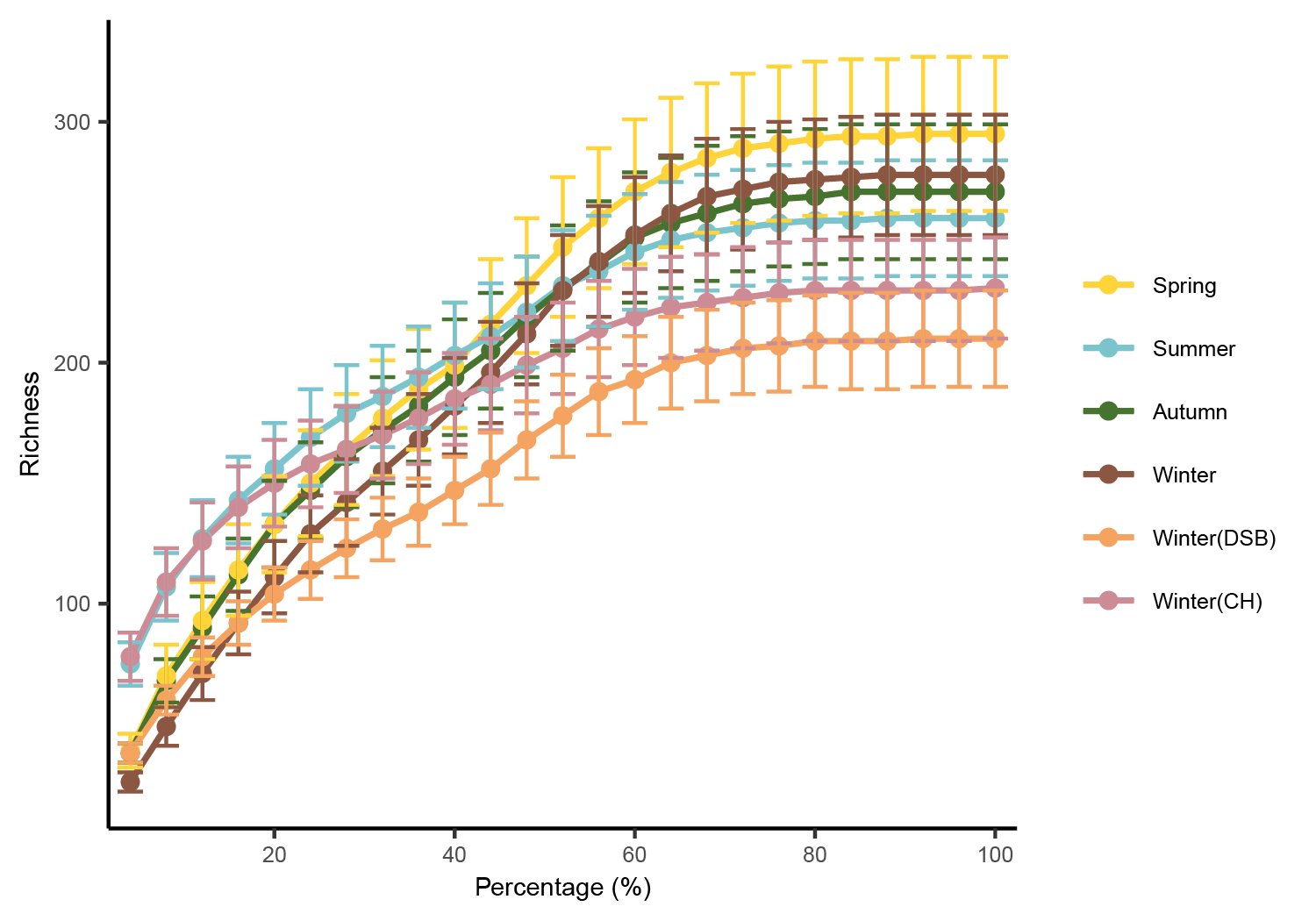


**Figure S2** Coverage of members in the microbiota by the black neck crane.


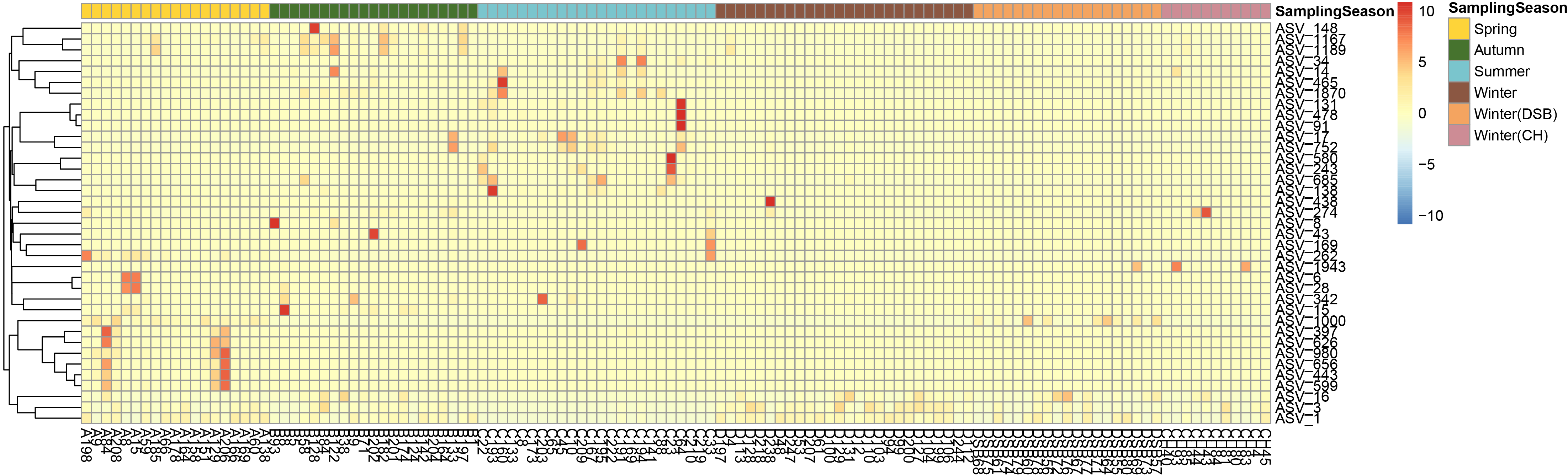


**Figure S3** The relative abundance of 36 ASVs in the gut microbiota was predicted using a random forest model across training set samples.


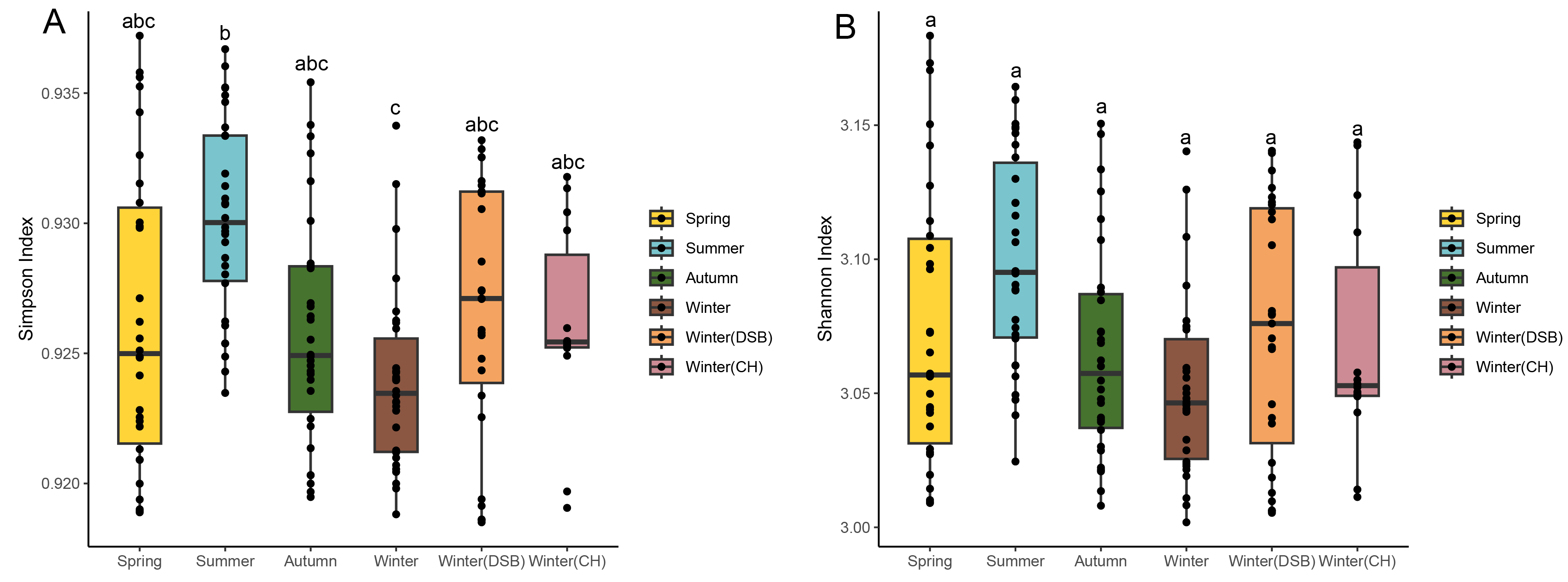


**Figure S4** Alpha diversity of the gut microbiota function was measured using (A) Simpson and (B) Shannon indices.
